# Doxorubicin treatment modulates chemoresistance and affects the cell cycle in two canine mammary tumour cell lines

**DOI:** 10.1186/s12917-020-02709-5

**Published:** 2021-01-18

**Authors:** Michela Levi, Roberta Salaroli, Federico Parenti, Raffaella De Maria, Augusta Zannoni, Chiara Bernardini, Cecilia Gola, Antonio Brocco, Asia Marangio, Cinzia Benazzi, Luisa Vera Muscatello, Barbara Brunetti, Monica Forni, Giuseppe Sarli

**Affiliations:** 1 Department of Veterinary Medical Sciences, University of Bologna, Ozzano dell’Emilia, Italy; 2 Department of Veterinary Sciences, University of Turin, Grugliasco, Italy

**Keywords:** Canine mammary tumour, Cell line, Chemoresistance, Doxorubicin, P-glycoprotein, BCRP, Cell cycle, p53, Telomerases, Proliferation index

## Abstract

**Background:**

Doxorubicin (DOX) is widely used in both human and veterinary oncology although the onset of multidrug resistance (MDR) in neoplastic cells often leads to chemotherapy failure. Better understanding of the cellular mechanisms that circumvent chemotherapy efficacy is paramount. The aim of this study was to investigate the response of two canine mammary tumour cell lines, CIPp from a primary tumour and CIPm, from its lymph node metastasis, to exposure to EC50_(20h)_ DOX at 12, 24 and 48 h of treatment. We assessed the uptake and subcellular distribution of DOX, the expression and function of P-glycoprotein (P-gp) and Breast Cancer Resistance Protein (BCRP), two important MDR mediators. To better understand this phenomenon the effects of DOX on the cell cycle and Ki67 cell proliferation index and the expression of p53 and telomerase reverse transcriptase (TERT) were also evaluated by immunocytochemistry (ICC).

**Results:**

Both cell lines were able to uptake DOX within the nucleus at 3 h treatment while at 48 h DOX was absent from the intracellular compartment (assessed by fluorescence microscope) in all the surviving cells. CIPm, originated from the metastatic tumour, were more efficient in extruding P-gp substrates. By ICC and qRT-PCR an overall increase in both P-gp and BCRP were observed at 48 h of EC50_(20h)_ DOX treatment in both cell lines and were associated with a striking increase in the percentage of p53 and TERT expressing cells by ICC. The cell proliferation fraction was decreased at 48 h in both cell lines and cell cycle analysis showed a DOX-induced arrest in the S phase for CIPp, while CIPm had an increase in cellular death without arrest. Both cells lines were therefore composed by a fraction of cells sensible to DOX that underwent apoptosis/necrosis.

**Conclusions:**

DOX administration results in interlinked modifications in the cellular population including a substantial effect on the cell cycle, in particular arrest in the S phase for CIPp and the selection of a subpopulation of neoplastic cells bearing MDR phenotype characterized by P-gp and BCRP expression, TERT activation, p53 accumulation and decrease in the proliferating fraction. Important information is given for understanding the dynamic and mechanisms of the onset of drug resistance in a neoplastic cell population.

**Supplementary Information:**

The online version contains supplementary material available at 10.1186/s12917-020-02709-5.

## Background

The ability of cancer cells to circumvent the effect of structurally unrelated chemotherapeutic agents is defined as multidrug resistance (MDR) [1–4]. One of the most efficient MDR mechanism is the overexpression of ATP-binding cassette (ABC) transporters by neoplastic cells that can remove substrates by pumping them out of the cell. Modifications of the expression and activity of ABC proteins can lead to an increased pumping out of drugs from the intracellular compartment and therefore to a reduction of the effective concentration of the drug in cancer cells [5, 6]. In human medicine, P-glycoprotein (P-gp), encoded by the *ABCB1* (or *MDR-1*) gene and the Breast Cancer Resistance Protein (BCRP), encoded by the *ABCG2* gene, are two of the many ABC transporters often associated with the MDR phenotype [1, 7]. P-gp and BCRP efflux pumps are often co-expressed on the lipid bilayer of cellular plasma membrane but can also be found at the membranes of intracellular organelles where drugs can be segregated [8–11].

One of the most used antineoplastic agents in both human and veterinary medicine is the anthracycline Doxorubicin (DOX). DOX exerts its effects by multiple mechanisms including the production of free radicals, intercalation into DNA strands and inhibition of topoisomerases I and II, causing damage to DNA, resulting in the activation of caspases, which ultimately leads to apoptosis [12, 13]. Nevertheless, DOX is a substrate of both P-gp and BCRP, which can decrease its intracellular concentration therefore limiting antineoplastic action [14, 15]. Despite extensive clinical utilization, the mechanisms of action of DOX remain under intense debate and further understanding of DOX influence on cell biological events could lead to an improvement in the drug’s efficacy [12, 13, 16]. Nowadays, cancer cell lines are successfully used in many studies as an *in vitro* model to study cancer biology, molecular pathways and test the efficacy of anticancer drugs [17].

Mammary neoplasms are among the most common tumours in dogs and humans [18]. In recent decades, canine mammary tumours (CMTs) have been successfully used as a spontaneous model for breast cancer research and important progress has been observed in veterinary oncology concerning the treatment and knowledge of this disease [19–21].

P-gp and BCRP expression in CMTs has been demonstrated using techniques able to detect their presence at the subcellular level [22–27], however studies investigating the functionality of the pumps with regard to the chemotherapeutic exposure are still incipient in the dog [28–30].

The aims of the present study were: (1) to investigate the MDR mechanism associated with DOX treatment on two CMTs cell lines, comparing the expression of P-gp, BCRP, tumour protein p53 (p53), the catalytic subunit of telomerase, telomerase reverse transcriptase (TERT) and the proliferation index Ki67 between standard condition and exposure to DOX treatment, and (2) to establish a repeatable *in vitro* model that allows to evaluate the *in vivo* chemotherapeutic drugs effects.

## Results

### Cell viability and Doxorubicin hydrochloride treatment

**Fig. 1:**
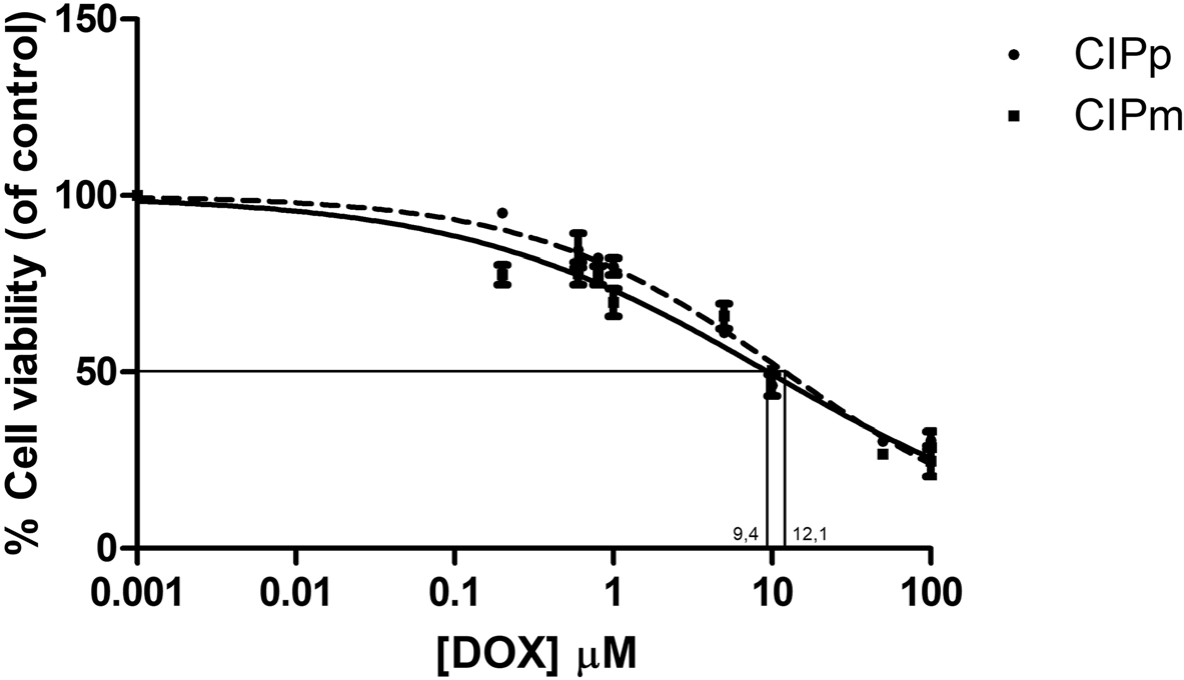
Effect of DOX on CIPp and CIPm cell viability. DOX impairs cell viability of canine mammary carcinoma cell lines, CIPp (dotted line) and CIPm (continuous line). Cells were treated with increasing concentrations of DOX for 20 h. The values for EC50_(20h)_ were normalized to the control cell lines (untreated) evaluated in the same culturing conditions. Dose-response curves represent mean ± s.e.m. from three independent experiments, each performed in quadruplicate. EC50_(20h)_ values were calculated using nonlinear regression curve by Prism 7 software (GraphPad San Diego, CA, USA)

Population doubling times (DT) were very similar in the two cell lines: 23 h and 17 min and 20 h and 29 min in CIPp and CIPm, respectively. The effect of DOX treatment on CIPp and CIPm viability was evaluated using the MTT assay. The cell lines had very similar sensitivity to DOX. The EC50 values at 20 h [EC50_(20h)_] were 12.08 μM and 9.431 μM for CIPp and CIPm, respectively. The cell viability values, compared to the various concentrations of chemotherapy treatment, are shown in Fig. 1.

### Doxorubicin-associated fluorescence evaluation

**Fig. 2:**
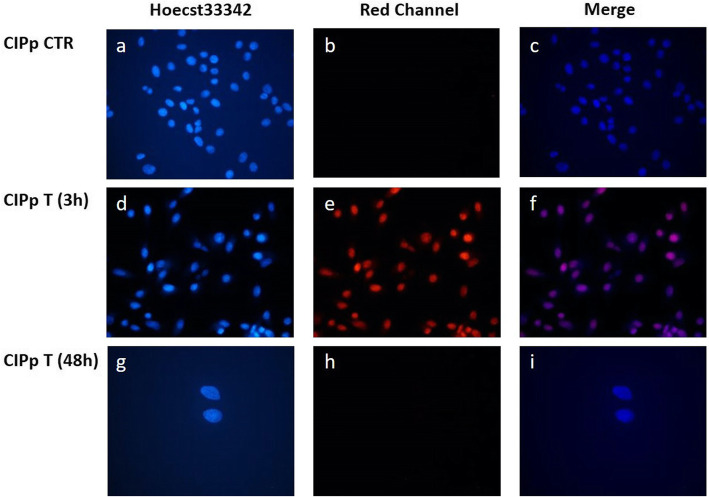
Doxorubicin-associated fluorescence in CIPp. DOX in CIPp control cells (CTR) and after 3 h and 48 h of EC50_(20h)_ treatment. Nuclei were stained with Hoechst33342 in blue (**a**, **d** and **g**). DOX red fluorescence in **b**, **e** and **h**. The merge images (**c**, **f** and **i**) containing the blue fluorescence of the nuclei and the red fluorescence of DOX

**Fig. 3:**
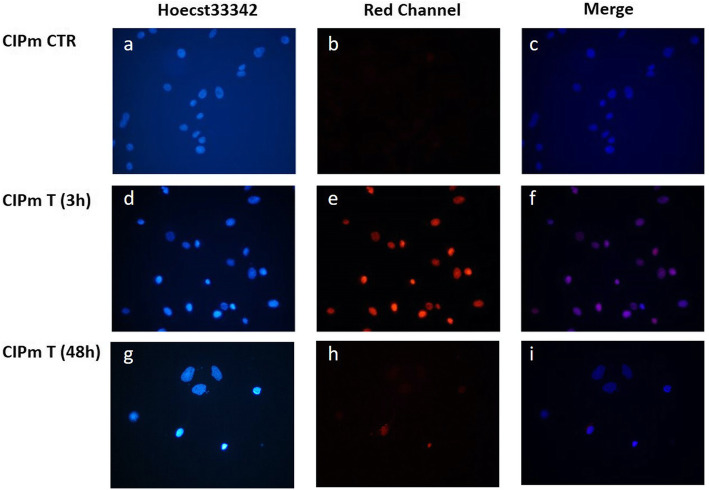
Doxorubicin-associated fluorescence in CIPm. DOX in CIPm control cells (CTR) and after 3 h and 48 h of EC50_(20h)_ treatment. Nuclei were stained with Hoechst33342 in blue (**a**, **d** and **g**). DOX red fluorescence in (**b**, **e** and **h**). The merge images (**c**, **f** and **i**) containing the blue fluorescence of the nuclei and the red fluorescence of DOX

By fluorescence microscopy we observed the blue fluorescence of Hoecst33342 in all nuclei of both cell lines, as well as a bright red fluorescence of DOX in the treated cells. In both CIPp and CIPm, after 3 h of treatment, almost all cells have internalized DOX and are therefore intensely colored red as shown in Figs. 2 and 3, respectively. The superimposition of the images highlights how the drug concentrates in the nucleus (Figs. 2f and 3f). At 48 h, all these surviving cells were unstained because they have extruded DOX (Figs. 2i and 3i).

### Cell cycle analysis by flow cytometry

We investigated the effect of DOX EC50_(20h)_ at 3, 6, 12 and 20 h on CIPp and CIPm distribution in the cell cycle phases.

**Fig. 4:**
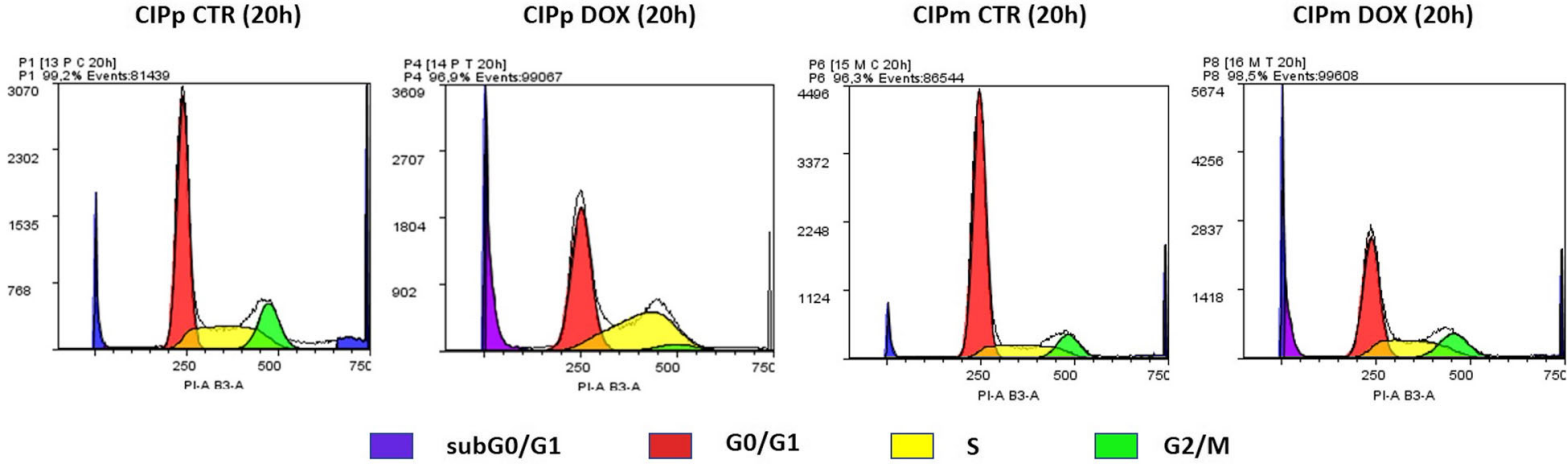
Dean-Jett-Fox Univariate cell cycle analysis by Flow Cytometry. Fluorescence of the PI-stained cells was measured using MACSQuant® Analyzer10 and analyzed by Flowlogic software (Miltenyi Biotec, Bergisch Gladbach, Germany). 2 × 10^5^ cells were examined for each sample and experiment was repeated three times. Representative DNA content frequency histograms in CIPp and CIPm treated for 20 h with DOX EC50_(20h)_ and in CTR CIPp and CIPm at the same time point

CIPp, treated with EC50_(20h)_ DOX showed a gradual increase of cells in the S (DNA synthesis) phase compared to the CTR (Fig. 4 and Additional file 1). Increase in S phase was associated with a progressive decrease of cells in G2/M (mitosis) phase and, at 20 h, there were no cells in G2/M (Fig. 4). The cell cycle underwent therefore an arrest at the S phase because of DOX treatment. Besides, the hypodiploid sub G0/G1-peak constantly increased reaching 21.76% ± 5.42% at 20 h in treated CIPp compared to 5.92% ± 2.61% in CTR cells (Fig. 4). Sub-G0/G1 peak was composed by apoptotic cells and by cells that had already lost their DNA by shedding apoptotic bodies, cellular fragments containing pieces of chromatin, broken nuclei, chromosomes and cellular debris [31].

CIPm did not show any severe increase in the S phase at any time point compared to the untreated controls. However, similarly to CIPp, also CIPm as well presented a gradual increase of the hypodiploid peak in DOX treated cells compared to CTR ones (18.63% ± 5.96% vs 3.52 ± 1.68%) (Fig. 4 and Additional file 2).

### *P-gp* and *BCRP* gene expression after treatments with Doxorubicin

**Fig. 5:**
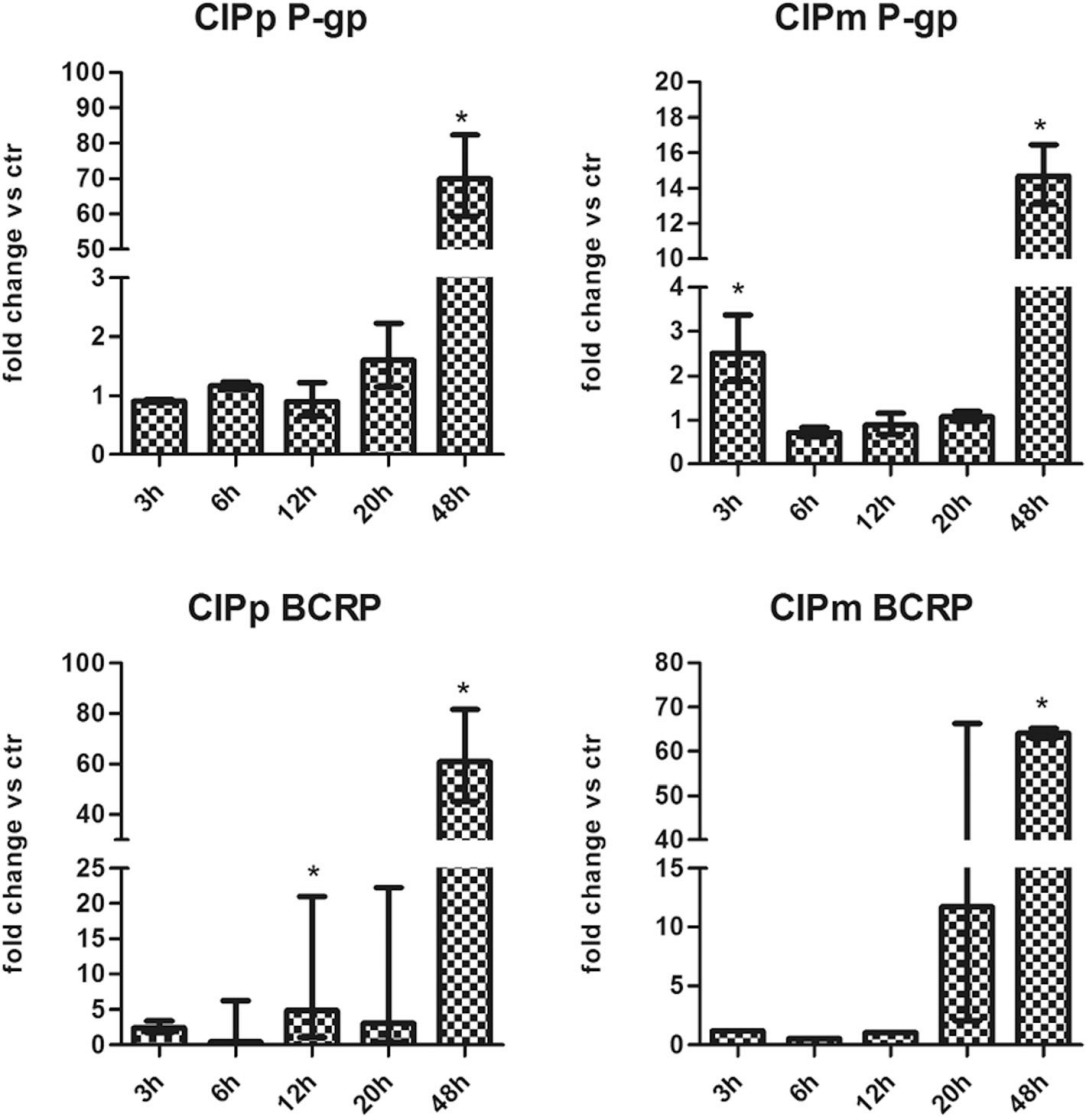
*P-gp* and *BCRP* gene expression in CIPp and CIPm treated with DOX EC50_(20h)_. Gene expression is calculated with the formula of 2 ^–ΔΔCt^ and shows the fold of change of treated cells vs their relative controls. The bars represent the minimum and maximum expression range of the genes. Asterisk indicates statistically significant differences (*p* < 0.05) calculated with the Student’s *t* test (comparing treated vs ctr at the same time points)

CIPp and CIPm were treated with the EC50_(20h)_ dose of DOX and the *P-gp* and *BCRP* gene expression profiles were measured by quantitative Real Time PCR (qRT-PCR) at different treatment times (3, 6, 12, 20, 48 h). *BCRP* and *P-gp* were detectable in all samples. A significant increase of both *P-gp* and *BCRP* mRNA expression level is evident at 48 h treatment time point for both CIPp (*p* = 0.0009 and *p* = 0.0132, respectively) and CIPm (*p* = 0.0013 and *p* = 0.0209, respectively) vs controls (Fig. 5). In addition, in CIPp at 12 h *BCRP* expression was significantly higher in the DOX treated and at 3 h *P-gp* expression of CIPm was significantly higher in the DOX treated cells vs controls.

### Antibodies validation by Western Blotting

Western Blot analysis was performed to investigate anti-P-gp (clone C494) and anti-BCRP (clone BXP-21) antibodies specificity for dog antigens. Dog liver tissue expressed a single band specific for P-gp and BCRP at the expected molecular weight of 140 kDa and 70 kDa, respectively (see Additional file 3). Anti-p53, -TERT and -ki67 (Mib1) antibodies specificity for dog tissues had been assessed in previous studies [32–35].

### Immunocytochemistry on chamber slides treated with Doxorubicin hydrochloride versus untreated

P-gp, BCRP, p53, TERT expression and the Ki67 proliferation index were evaluated in CIPp and CIPm by immunocytochemistry (ICC) at 12, 24, 48 h of treatment with the EC50_(20h)_ dose of DOX and compared to the corresponding control cultures. Extremely high density of cells was present at 48 h culture for the untreated culture; for the 48 h treatment group, in which cells were less numerous due to the longer treatment exposure, all cells present in the chamber were examined. ICC results and statistical comparisons are reported in Additional file 4.

**Fig. 6:**
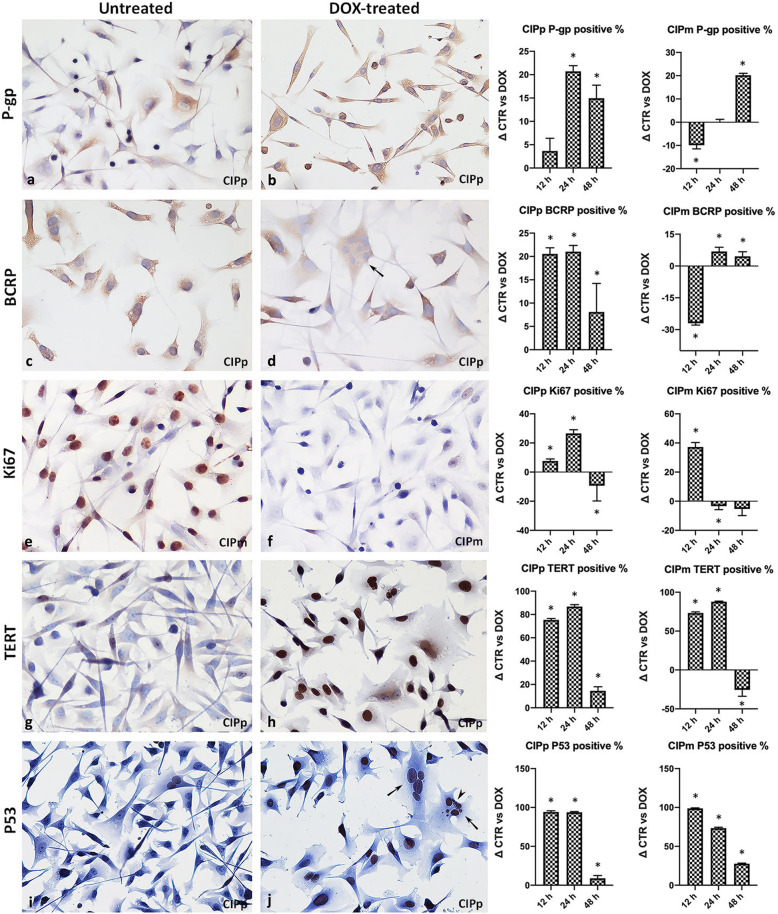
Immunocytochemistry on chamber slides treated with Doxorubicin. Untreated (**a**, **c**, **e**, **g**, **i**) and DOX-treated (**b**, **d**, **f**, **h**, **j**) CIPp and CIPm ICC with graphs representing the % differences (Δ) in positivity between the controls and the DOX-treated cells for each antibody. Cell percentages were averaged over triplicate samples, and the data are expressed as the mean ± SD, asterisk represent a significant difference (*p* < 0.05) between controls and treated cells by two-tail Fisher exact test. ICC at 24 h treatment for P-gp in untreated (**a**) and DOX-treated (**b**) CIPp cell line. ICC for BCRP in untreated (**c**) and DOX-treated (**d**) CIPp cell line. ICC for Ki67 in untreated (**e**) and DOX-treated (**f**) CIPm cell line. ICC for TERT in untreated (**g**) and DOX-treated (**h**) CIPp cell line. ICC for p53 in untreated (**i**) and DOX-treated (**j**) CIPp cell line; multinucleated cells, indicated by the black arrow, are shown in (**d**) and (**j**) and cells with micronuclei, indicated by black arrowhead, are shown in (**j**). Figures at 200x magnification

Positive staining for P-gp and BCRP was localized at the cell membrane segments, frequently on cytoplasmic vacuoles membranes and less intensely as dispersed granular staining in the cytoplasm (Fig. 6). The percentage of P-gp positive cells were increased at all time points in CIPp with a statistically significant increase at 24 and 48 h of treatment (*p* < 0.0001). CIPm showed a significantly increased percentage of P-gp positive cells at 48 h (*p* < 0.0001) and a mild increase at 24 h treatment. A significant increase in the percentage of BCRP positive cells was seen at all treatment time points for CIPp (*p* < 0.0001 at 12 and 24 h; *p* = 0.0151 at 48 h) and at 24 h and 48 h treatment for CIPm (*p* = 0.0103 and *p* = 0.0413, respectively) compared to the controls. The treatment with DOX was overall associated with an increase in the percentage of P-gp and BCRP positive cells in the culture by ICC (Fig. 6b and d). The fraction of cells replicating in the populations was evaluated by the Ki67 proliferation antigen as specific nuclear staining in Ki67-positive cells (Fig. 6e and f). At 12 h both cell lines revealed a significantly higher proliferative activity in the DOX treated cultures compared to untreated controls. At 24 h CIPm showed a mild decrease of the proliferation index, while CIPp proliferation index was significantly increased compared to the respective untreated controls (*p* = 0.0034). At 48 h of treatment a decrease in the cellular proliferative fraction was present for both cell lines, but statistically significative only for CIPp (*p* = 0.0118).

P53-positive cell showed specific nuclear staining, while cellular TERT-positivity was represented by nuclear and/or granular cytoplasmic staining (Fig. 6g–j).

A striking significant increase was observed for both p53 and TERT expression, compared to controls at each time points (*p* < 0.0001 at each time point) except for CIPm at 48 h treatment when a higher percentage of TERT positive cells was detected in untreated cells.

Cells treated with EC50_(20h)_ of DOX demonstrated an increase in cell size for both cell lines and, for CIPp only, the presence of multinucleated cells, often in association with karyomegaly and multiple micronuclei (Fig. 6(d), (h) and (j)).

### MDR direct dye efflux assay

**Fig. 7:**
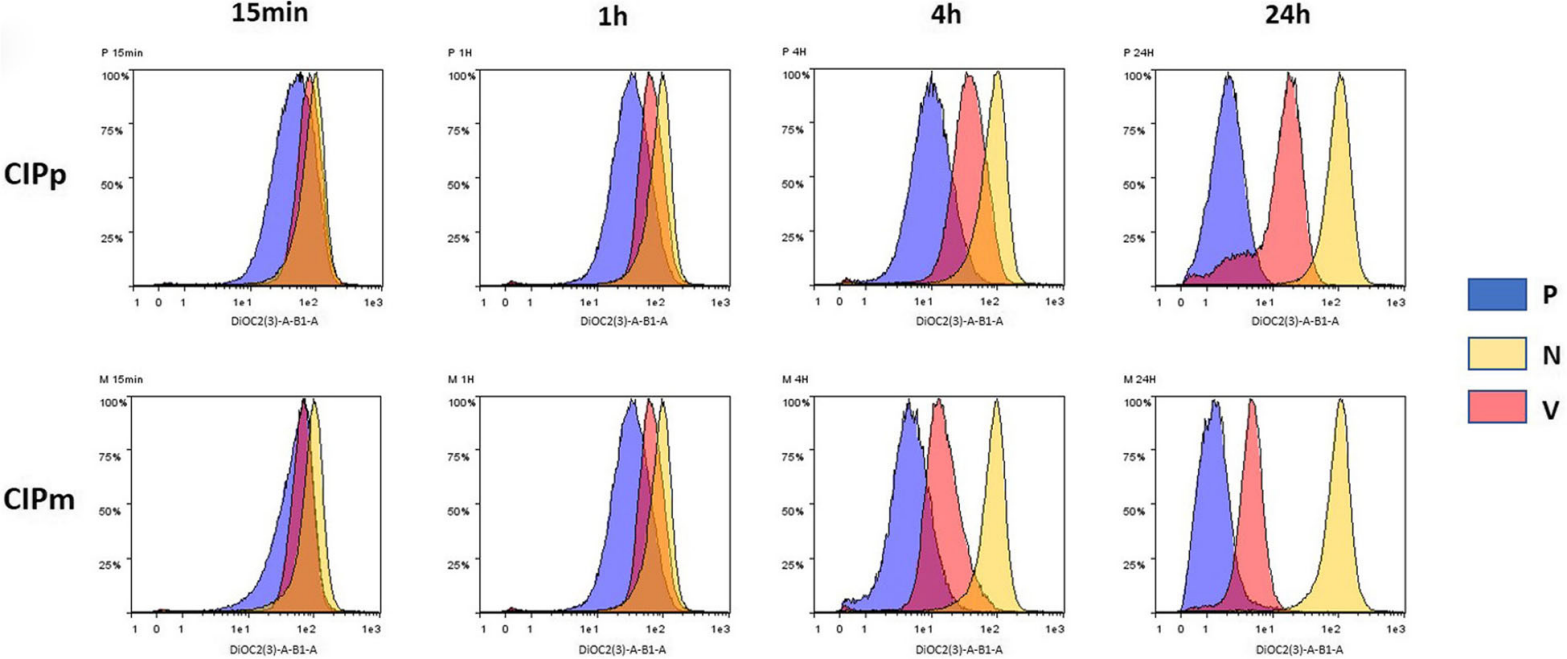
Multidrug Resistance Direct Dye Efflux Activity in CIPp and CIPm. Cells were loaded with DiOC2(3) and incubated at 4 °C, or at 37 °C in the presence or absence of Vinblastine for 15 min, 1, 4 and 24 h. After washing and PI staining, cells were analyzed by flow cytometry and dead cells were excluded from analysis. Cells incubated at 4 °C (negative control - N) exhibit high fluorescence, as P-gp and BCRP are inactive at low temperatures and the cells retain the dye. Cells incubated at 37 °C (positive control - P) have a progressively lower fluorescence over time, as P-gp and BCRP are active and cells efflux the dye. Cells incubated at 37 °C in the presence of Vinblastine (V) that specifically effluxes via P-gp, have higher fluorescence compared to control cells, as Vinblastine competes with the dye for efflux by P-gp

The *MDR Direct Dye Efflux Assay* was performed to evaluate the functionality of P-gp and BCRP expressed by CIPp and CIPm. In both CIPp and CIPm the fluorophore efflux was evident at each time point becoming almost complete at 24 h (Fig. 7). Vinblastine, a well-known substrate for P-gp pumps, exerts a selective competitive inhibitory effect and was used as an internal control. In both CIPp and CIPm, P-gp efflux capacity was dampened by the treatment with Vinblastine at all time points; CIPm were less affected by Vinblastine treatment as evidenced by the difference (Δ) in MFI between positive control cells and Vinblastine treated ones (P-V), CIPm were able to extrude a larger dye quantity than the CIPp in any case except at 1 h (Fig. 7). Representative histograms of the MDR Direct Dye Efflux Activity in CIPp and CIPm are displayed in Fig. 7, where a peak shoulder is observed at 24 h in Vinblastine treated CIPp cells; this peak may be related to the presence of two cell populations, a smaller one rich in channels, able to efflux a quantity of dye similar to positive control cells even in presence of the inhibitory competitor, and a larger cell population in which the efflux is clearly limited by Vinblastine presence (Table 1).

**Table 1:** Multidrug Resistance Direct Dye Efflux Activity in CIPp and CIPm

	CIPp		CIPm	
	Δ (N-P) MFI	Δ (P-V) MFI	Δ (N-P) MFI	Δ (P-V) MFI
**15 min**	43.87 ± 5.74	−33.34 ± 5.68*	41.55 ± 5.69	− 14.49 ± 5.38*
**1 h**	60.15 ± 5.81	−34.09 ± 5.33	59.75 ± 6.15	−29.15 ± 6.11
**4 h**	81.32 ± 5.25	−28.25 ± 6.01*	86.71 ± 5.13	−9.70 ± 5.08*
**24 h**	101.06 ± 4.98	− 13.58 ± 5.12*	101.47 ± 4.85	−3.49 ± 2.54*

Differences (Δ) between median fluorescence intensity (MFI) values: ΔN-P (negative controls at 4 °C - positive controls at 37 °C) represents the amount of fluorescent dye effluxed at each time point, ΔP-V (positive controls at 37 °C - cells treated with Vinblastine at 37 °C) represents the reduction of dye effluxed in presence of Vinblastine in CIPp and CIPm. Asterisk indicates a significative difference between the two cell lines in the same condition (Student’s t test *p* < 0.05)

## Discussion

### P-gp and BCRP changes at gene expression and cellular phenotype levels after exposure to Doxorubicin

The administration of DOX significantly enhanced the transcription of *P-gp* and *BCRP* genes, presumably associated to a functional need of efflux pumps in cells targeted by DOX [28, 36]. CIPp, the cell line established from the primary CMT, at 48 h showed increase in the gene expression of both *P-gp* and *BCRP*. CIPm, cells forming the lymph node metastases, showed a greater increase of *BCRP* than *P-gp*, compared to controls.

At ICC, the percentage of cells immunolabeled by P-gp and BCRP was highly variable. CIPp manifested an increase in the expression of both the transporters compared to the controls at any time points, whereas in CIPm the percentage of P-gp and BCRP positive cells was higher at 24 h for BCRP and, at 48 h of treatment, for P-gp. The difference in the results between ICC and qRT-PCR can be explained by the fact that at early treatment time points (12 and 24 h) DOX-sensitive cells died while the cells bearing intrinsic DOX resistance remained viable and could be stained by ICC. Instead, qRT-PCR was able to detect an increase in the mRNA transcript mostly evident at 48 h when cells subjected to treatment enhance their transcription machine in order to increase their ability to survive DOX treatment. Moreover, qRT-PCR is a quantitative method that detects the presence of a specific mRNA over the total mRNA in a cellular population, but it does not allow the detection of cellular diversity in the population; on the other hand ICC highlights single positive cells but not to permit the quantifications of the number of the pumps present in a single cells. The number of P-gp and BCRP positive cells by ICC, is not necessarily related to the amount of gene expression detected by qRT-PCR. The number of cells evaluated by ICC in the chamber slides at 48 h was extremely reduced by the EC50_(20h)_ DOX treatment, hence representing a bias for quantification of the pumps.

Intrinsic expression of P-gp and BCRP was shown in the two cell lines examined in this study. The treatment with DOX demonstrated that cells that were able to survive had a higher expression of both P-gp and BCRP thus they escaped the drug action through MDR related mechanisms [5, 37].

Drug resistance to vinblastine in CMTs cell lines is documented [30] and a high expression of P-gp [29] and BCRP [28] in CMT cell lines led to the conclusion that the use of DOX could lead to chemoresistance in treated bitches [28].

In the present study ICC has been a valuable technique for assessing the subcellular localization of P-gp and BCRP which were identified both at the plasma membrane and in the cytoplasm of the cells, lining cytoplasmic vacuoles. The expression of both ABC-transporters has been documented at membranous level, where they exert their function of efflux pumps, extruding drug from the nucleus and cytoplasm [5, 38, 39], while in the cytoplasmic cellular compartment where they are found on cytoplasmic lysosomal vacuoles, known as drug safe houses, which have the function of segregating the drug preventing its action into the nucleus [10, 11, 40–42].

Another interesting finding at ICC was the presence of a substantial fraction of cells showing karyomegaly, micronuclei and multinucleated cells in treated CIPp especially. This finding can be associated with a DNA damage due to DOX treatment that increases ploidy and severe nuclear anomalies of cancer cells including giant cells, polyploid and polymorphism, comprising multinucleated cells and micronuclei that have been reported as an indicator of genetic instability [43–46].

### Effects of Doxorubicin on cell cycle progression and cellular proliferation – Ki67 index, p53 and TERT

In the present study DOX exposure showed an important effect on both CIPp and CIPm affecting cellular distribution in the cell cycle phases. The cytotoxic action of DOX resulted in cell cycle arrest in S phase for CIPp complemented by cellular death increase. On the contrary, CIPm did not arrest at cellular checkpoints, but the reduction of cellular percentage in G0/G1 went on parallel with cellular death increase. The absence of the arrest at S phase in CIPm could be interpreted as a loss of the cellular checkpoints associated with the progression of the tumour from primary (CIPp) to metastatic (CIPm). Besides, changes in Ki67 labeling index measured during treatment are reportedly to be a superior predictor of response than similar measurements taken prior to treatment [47]. DOX is known for influencing the cell cycle and proliferative activity with different effect associated to the cell type and dose of DOX itself [48] and can influence the proliferative activity in an heterogeneous way depending on the dose and the cell line as well by different gene expression [49].

The most evident effects of DOX treatment on the cellular phenotype were the striking increase in the percentage of p53 and TERT positive cells for both cell lines when treated with DOX EC50_(20h)_ at all time points. In fact, DOX has multiple mechanism of action involving, in some cases, the activation of p53 [48]. Moreover, the genetic instability, which is the consequence of DOX administration and loss of wild type p53 function, has been suggested as the property which allows highly malignant cells to amplify the *MDR-1* gene and thus become resistant to a wide spectrum of cytotoxic drugs [50].

Hence, an important information was that in both untreated cell lines p53 is functional/not mutated and that DOX treatment could activate its function. P53 activation conceivably mediated the cell cycle arrest in the S phase in CIPp and cellular death in CIPm. A functional p53 can represent a protective mechanism in a neoplastic population treated by DOX, in fact the arrest of the cell cycle can lead to p53-mediated activation of reparative processes in neoplastic cells that could therefore survive the treatment and be able to regrow upon chemotherapy withdrawal [49, 51, 52]. P53 function has an important role in chemotherapy efficacy, mediating apoptosis of neoplastic cells targeted by many drugs [53–57].

At 48 h treatment an increase of the percentage of p53 positive cells is seen in untreated cell lines, probably because of hyper-confluence and deficiency of nutrients of the cell culture [58].

A significant increase in TERT activation and expression, both in nuclei and cytoplasm, is another finding in DOX treated cell lines. These can be explained by an attempt of neoplastic cells to protect themselves from the damage induced by DOX, conferring drug resistance. Telomerase is the ribonucleoprotein responsible for *de novo* synthesis and maintenance of telomeres, and its activity is predominantly observed in cancer cells [59, 60]. It is well known that a decreased expression or inhibited activity of telomerase in cancer cells is accompanied by an increased sensitivity to some drugs (e.g., DOX, cisplatin, or 5-fluorouracil) [55], however, the mechanism of the resistance resulting from telomerase alteration remains elusive [59]. Telomerase can translocate from the nucleus into the mitochondria to protect the cells from apoptosis mediated by ROS stress, being ROS production a well-known mechanism of damage from DOX to neoplastic cells [59, 60]. TERT can prevent cell death by elongating and rebuilding the telomeres and by increasing TERT concentration in the mitochondria preventing apoptosis, influencing therefore the efficacy of the treatment [54, 59, 61, 62]. The minimal set of changes necessary to obtain tumorigenic drug-resistant cells was found to be expression of telomerase and inactivation of p53 and pRb [63], thus as the pathways inactivated or activated in malignant neoplasms can also confer the ability to acquire drug resistance as was observed also in our populations of cells.

### Internalization and extrusion of Doxorubicin at different timepoints

The exact mechanism of action of DOX is complex and still unclear. To exert its function, a drug must reach its intracellular target that, in the case of DOX, is primarily the nucleus [13, 15, 64]. After 3 h of treatment, DOX reached its target in both CIPp and CIPm while at 48 h treatment the drug was extruded from the few surviving cells. The Multidrug Resistance Direct Dye Efflux Assay showed that P-gp and BCRP channels were active in both cell lines and able to extrude the substrate in 24 h. Overall, CIPm were more efficient at extruding the exogenous compound compared to CIPp. Being CIPm derived from metastatic tumour this finding could be associated to an increased drug resistance capacity of the metastatic cells compared to their primary counterpart, a phenomenon already reported both *in vivo* and *in vitro* [65–68]. Since metastasis is the major cause of cancer death, it is important to address the effect of chemotherapy on metastasis by accurately establish the response of a cell population to the drug to be administered to ensure that the desired effect will be observed both in the metastases and primary tumours [44, 48, 68]. Chemotherapy can radically increase the speed of clonal evolution and lead to new malignant and resistant clones that can cause tumour metastasis. Several aspects of metastatic clones are distinct from primary tumour formation and could influence drug development [69, 70].

The qRT-PCR and ICC positively correlate with the test evaluating the transporters functionality (i.e. direct fluorescence of DOX). In fact, at 48 h the cells that survived treatment showed a complete extrusion of DOX, therefore carrying a phenotype particularly rich in P-gp and BCRP canals. The Multidrug Resistance Direct Dye Efflux Assay showed similar functionality of P-gp and BCRP in CIPp and CIPm in basal conditions, while CIPm showed a decreased sensitivity to the competitive inhibition of the P-gp canals by Vinblastine. CIPm, derived from the metastatic carcinoma, has more efficient P-gp canals, or canals of other types (i.e. MRP-1, LRP) compared to the primary tumour. Moreower, the extrusion assay detected a more complex response in CIPp, with the evidence of different sub-populations of neoplastic cells. A fraction of cells in the primary tumour were more effective in extruding Vinblastine and probably constitute the highly efficient population of CIPm, in the metastatic tumour [68, 71, 72].

Severe effect on cell viability were seen at 48 h DOX treatment, causing fewer cells available for examination, therefore the results related to this time point should be interpreted with caution.

## Conclusions

The present study investigated the relationship between cancer cells chemosensitivity, the neoplastic phenotype and the subcellular distribution of the chemotherapeutic drug DOX in two CMT cell lines CIPp, originated from a primary mammary tumour, and CIPm, initiated from the corresponding lymph node metastases. These cell lines were a valuable model to study the effect of chemotherapy treatments and to investigate the mechanisms associated with cell-survival and death in a subpopulation of cells subjected to chemotherapy. Changes in the cell cycle, p53 expression and TERT activation are interlinked with the modulation of P-gp and BCRP drug transporters and are associated to drug resistance. These findings provide valuable information for revealing the correlation between chemoresistance, phenotypic changes and proliferation dynamics in the neoplastic populations that might significantly contribute to reach over the time to neoplastic survival despite chemotherapy.

## Methods

### Cell culture

The canine mammary carcinoma cell lines, CIPp and CIPm, were kindly provided by prof. Takayuki Nakagawa from the University of Veterinary Medicine of Tokyo, Japan [73]. They were both obtained from a tubular carcinoma of a ten-years-old ShihTzu female dog; CIPp were derived from the primary tumour mass at mammary gland, whereas CIPm from the metastasis of the primary tumor to the regional lymph node. Both the primitive tumour and its metastasis cell lines were first isolated and established by Uyama et al. (2006) [74] showing that both the cells resulted negative for PR and ER receptors and positive for HER2 protein. Cultured CIPp and CIPm cells were maintained in RPMI 1640 with Glutamax medium supplemented with 10% FBS, 5 mg/L gentamicin sulphate, 6 mg/L fungizone (complete medium) and incubated at 37 °C in a humidified atmosphere of 5% CO_2_. Reagents were purchased by Gibco-Life Technologies (Carlsbad CA, USA). Cells were routinely cultured in T25 tissue culture flasks (T 25-Falcon, Beckton-Dickinson, Franklin Lakes, NJ, USA). Successive experiments were conducted in 96-well plates (MTT test), 24-well plates (RNA extraction), 8-slide chambers (DOX-associated fluorescence evaluation and immunocytochemistry) and T25 tissue culture flasks (cell cycle analysis and Multidrug Resistance Direct Dye Efflux Assay). Doxorubicin chlorohydrate (2 mg/ml) was supplied by Teva Pharmaceutical Industries (Petah Tiqwa, Israel).

### MTT assay and EC50 determination

Cells (3 × 10^3^/well) were plated into 96-well plates, allowed to attach for 6 h, and treated with 0 μM (control), 0.2, 0.6, 0.8, 1, 5, 10, 50, 100 μM DOX for 20 h in a humidified CO_2_ incubator. Drug was dissolved in complete medium. Mitochondrial activity, as index of cell viability, was evaluated by (3-(4,5-dimethylthiazol-2-yl)-2,5-diphenyltetrazolium bromide or MTT test (Sigma-Aldrich, St. Louis, MO, USA) following manufacturer’s instructions. Briefly, samples were incubated with MTT solution (0.5 mg/mL in PBS) for 2 h, formazan crystals were dissolved in 0.1 mL/well of MTT Solubilization Solution and absorbance spectrophotometrically measured at 570 nm using the Infinite® F50/−Robotic Absorbance microplate readers (Tecan Life Sciences, Männedorf, Switzerland).

### Doxorubicin-associated fluorescence evaluation

Due to its fluorescence properties and the use of a blue fluorescent dye Hoechst33342 labelling the nucleus, the intracellular distribution of DOX could be observed by the fluorescence microscope [75]. Cells (15 × 10^3^/chamber) were plated in 8-chamber slides with 500 μl of complete medium and allowed to attach for 24 h in a humidified CO_2_ incubator. Medium from each chamber was removed and cells treated with 0 μM (control) or EC50_(20h)_ DOX dose: 12.08 and 9.431 μM for CIPp and CIPm respectively. After 3 and 48 h of DOX treatment, medium was withdrawn, and cells were stained for 30 min a 37 °C with Hoechst33342 (Miltenyi Biotec) diluted 1:1000 in complete medium. Cells were washed twice with DPBS (Gibco-Life Technologies) then chambers were detached from slides. [Falcon™, Thermo Fisher Scientific, Waltham, Massachusetts, USA] Coverslips were mounted with Fluoroshield (Sigma-Aldrich) and images were obtained with an Eclipse E600 epifluorescence microscope (Nikon, Japan) equipped with a digital camera (Nikon, Japan).

### Cell cycle analysis by flow cytometry

CIPp and CIPm were seeded in T25-flask in complete medium and, when confluence reached approximately 70%, cells were treated with EC50_(20h)_ DOX dose: 12.08 and 9.431 μM for CIPp and CIPm respectively for 3, 6, 12 and 20 h in a humidified CO_2_ incubator. Untreated cells were employed as controls. At each time point cells were harvested and counted. Aliquots of 1 × 10^6^ cells were washed twice in 5 ml of PBS w/o Ca^2+^ and Mg^2+^ (Gibco-Life Technologies) and fixed overnight in 70% ice-cold ethanol (1 ml) added drop-by-drop with continuous vortexing. Then, the cells were washed with 10 ml of PBS and cellular pellet was incubated with 1 ml of staining solution containing 50 μg/ml of PI (Miltenyi Biotec, Bergisch Gladbach, Germany) and 100 μg/ml RNaseA/T1 (Thermo Scientific) in PBS for 30 min in the dark at room temperature (RT). Cell distribution in cell cycle phases was analyzed by MACSQuant® Analyzer10 and Flowlogic software (Miltenyi Biotec). Cellular events were discriminated from debris using forward (FSC-A) and side scatter (SSC-A). Doublets have been excluded for analysis by FSC-area and width (FSC-A/FSC-W). Dean-Jett-Fox Univariate Model was used to determine the percentage of the cell population in different phases of the cell cycle [76].

### RNA extraction and quantitative real time PCR (qRT-PCR) for P-gp and BCRP after treatments with Doxorubicin

CIPp and CIPm were seeded in a 24 wells plate (10^5^ cells/well) and exposed to DOX EC50_(20h)_ dose for different time (3, 6, 12, 20 and 48 h).

The total RNA was extracted using the TRI Reagent and the NucleoSpin RNA II kit. At the end of time and treatment, the cells were lysed using TRI Reagent (1000 μl/well) and mixed by vortex (30 s), then chloroform (200 μl) was added to the suspension and mixed well. After incubation at room temperature (10 min), the samples were centrifuged at 12000 x g for 10 min at + 4 °C and the aqueous phase was recovered. An equal volume (1.1; v.v) of ethanol (70%) was added and the resulting solution was applied to the NucleoSpin RNA Column. The RNA purification was performed according to the manufacturer’s instructions. The extracted RNA was quantified using the DeNovix DS-11 Spectrophotometer (DeNovix – Wilmington, DE, USA), and an A260/A280 ratio was used to evaluate RNA extraction quality. Five hundred nanograms of RNA were retrotranscribed to obtain cDNA in a 20 μL final volume, using an iScript cDNA synthesis kit. Quantitative PCR was carried out using a CFX96 thermocycler (Bio-Rad Laboratories). A master mix of the following reaction components was prepared in nuclease-free water to the final concentrations indicated: 0.2 μM forward primer, 0.2 μM reverse primer, and 1X iTaq Universal SYBR Green Supermix. Two microliters of cDNA were added to 18 μL of master mix. All samples were analyzed in duplicate. The amplification cycle for the real time PCR reaction is: 3 min at 95 °C, 40 cycles at 95 °C for 10 s and 60 °C for 30 s, followed by a melting step from 55 °C going up to 95 °C at a rate of 0.5 °C/5 s increment/cycle. The specificity of the amplified PCR products was confirmed by agarose gel electrophoresis and melting curve analysis.

Primers sequences of interested genes (*BCRP* and *P-gp*) and reference genes (glyceraldehyde 3-phosphate dehydrogenase, *GAPDH*; Ribosomal protein L32, *Rpl32*; Succinate dehydrogenase, *SDHA*) are reported in Table 2.

**Table 2:** Informations on primer sequences used for qRT-PCR analysis

Gene	Sequence (5′-3′)	PCR bp	AN	Reference
*P-gp*	*For* GCTTAACACCCGGCTCACAGAC	402	FJ617477.1	Pawlowski et al., 2013 [30]
	*Rev TAAGAAAGCGGCACCAATAGAAT*			
*BCRP*	*For* TTAGACTCCAGCACAGCAAATG	189	DQ222459.1	Present study
	*Rev* AACCCACTGACGCAAAGAAC			
*GAPDH*	*For* TGTCCCACCCCCAATGTATC	100	NM_001003142	Zannoni et al., 2020 [77]
	*Rev* CTCCGATGCCTGCTTCACTACCT			
*Rpl32*	*For* GGCACCAGTCAGACCGATATG	209	NM_001252169.1	Zannoni et al., 2020 [77]
	*Rev* GCACATCAGCACTTCAAG			
*Sdha*	*For* CGCATAAGAGCCAAGAAC	194	XM_535807	Zannoni et al.; 2020 [77]
	*Rev* CCTTCCGTAATGAGACAAC			

Real-time efficiency for interested genes was preliminarily evaluated by amplification of a standardized amount of cDNA, starting from 150 ng with subsequent 5 dilutions (75, 15, 3, 0.6 and 0.12 ng) derived from liver sample (positive control) (qPCR efficiency: *BCRP* 101%, *P-gp* 94.7%, data not shown).

The expression level of the interest gene (GI) was determined using the 2^-∆∆Ct^ method [78] in which ∆Ct = (Ct _interest gene_ – Ct _mean ref. genes_) and ∆∆Ct = ∆Ct _DOX group_ - ∆Ct _Control group_.

### Antibodies validation by Western Blotting

Anti-P-gp (clone C494) and anti-BCRP (clone BXP-21) antibodies were employed after their validation on canine tissues by Western Blot. For this purpose, canine liver (positive control) and muscle (negative control) tissue samples were collected, frozen in liquid nitrogen and stored at −80 °C until sample processing. Hundred milligrams of tissue were homogenized in 1 ml of SDS buffer (Tris-HCl, 62.5 mM; pH 6.8; SDS, 2%; and glycerol, 20%) supplemented with a protease inhibitor cocktail (Sigma-Aldrich). Total protein content was determined by Peterson’s Modification of Lowry Method using a Protein Assay Kit (Sigma-Aldrich). Ten and 30 μg of total proteins were separated on NuPage4–12% bis-Tris Gel (Thermo Scientific, Waltham, MA, USA) for 1 h at 165 V. The proteins were then electrophoretically transferred onto a nitrocellulose membrane by a semi-dry system (Trans Turbo Blot, Bio Rad, Hercules, CA, USA). Non-specific binding on nitrocellulose membranes was blocked with ECL Blocking Agent (GE Healthcare Life Sciences, Little Chalfont, Buckinghamshire, UK) in TBS-T20 (Tris Buffered Saline-0.1% Tween-20) for 1 h at RT. After blocking treatment, the membranes were incubated overnight at 4 °C with the primary antibodies anti-P-gp (clone C494, GeneTex, Irvine, CA, USA) and anti-BCRP (clone BXP-21, Merck Millipore, Darmstadt, DE) 1:800 and 1:1000 respectively in Blocking Agent. Anti-Alfa-tubulin antibody (TU-01, Thermo Scientific) was employed to reveal this housekeeping protein (1:500 in TBST-T20). After washes, the blot was incubated with an anti-mouse IgG, HRP-linked Antibody (Cell Signaling Technology, Danvers, MA, USA), at 1:10000 dilution in TBS-T20, 1 h at RT and then with an anti-biotin horseradish peroxidase (HRP)-linked antibody (Cell Signaling Technology), (1:100 dilution, 40 min at RT).

Immunoreactive bands were visualized using chemiluminescent substrate (Clarity Western ECL Substrate, Bio Rad), according to the manufacturer’s instructions. The intensity of the luminescent signal was acquired by Chemidoc Instrument (Bio Rad) and the apparent molecular weight of the resultant bands was analyzed by Quantity One Software (Bio-Rad). Western blot analysis of P-gp and BCRP revealed a single band of expected molecular weights, ~ 140 kDa and ~ 75 kDa respectively, in canine liver tissue (30 μg) but not in canine muscle tissue.

### Cell immunocytochemistry

Considering the generational time of the two cell lines (TCIPp = 23 h and 17 min; TCIPm = 20 h and 29 min), cells (20 × 10^3^ and 15 × 10^3^/chamber per CIPp and CIPm lines, respectively) were plated in 8-chamber slides (BD Falcon™ Chamber Cell Culture Slides; Coring Life Sciences, Bedford, MA, USA) with 500 μl of complete medium (Falcon™, Thermo Fisher Scientific, Waltham, Massachusetts, USA) and allowed to attach for 24 h in a humidified CO_2_ incubator.

The complete culture medium was removed. Fresh culture medium was added for the controls, instead culture medium added to DOX at a concentration of 1 μM was added for treated chambers. Chamber slides were incubated for 12, 24, 48 h at 37 °C at 5% CO_2_ to assess P-gp, BCRP, p53, TERT and Ki67 expression. The control medium was then removed from chambers, then washed in. Cells were fixed for 30 min in 4% formic aldehyde buffered at pH 7.2, followed by 3 washings in PBS.

Chamber slides were subjected to endogenous peroxidases inhibition (H_2_O_2_ 0.3% in demineralized water for 5 min). Chambers were incubated for 3 min with a solution of PBS at pH 7.2. Each well underwent a 45-min dark pre-incubation with a serum free blocking solution (Protein block serum free; Agilent Technologies, Santa Clara, CA, USA). Chamber slides were then incubated overnight at 4 °C with (1) mouse monoclonal anti-P-gp (clone C494, GeneTex, Irvine, CA, USA), diluted 1:1500 in 1% bovine serum albumin (BSA; Sigma-Aldrich) in PBS; with (2) mouse monoclonal anti-BCRP (clone BXP-21, Merck Millipore, Darmstadt, DE), diluted 1:800 in 1% BSA in PBS; with (3) mouse monoclonal anti-Ki67 (clone Mib1, Dako, Glostrup, DK), diluted 1:600 in 1% BSA in PBS; with (4) mouse monoclonal anti-p53 (clone Pab 240, BD Pharmingen, San Jose, CA), diluted 1:100 in 1% BSA in PBS; and with (4) anti-hTERT monoclonal antibody (clone 44F12, Novocastra, Newcastle upon Tyne, UK), diluted 1:50 in 1% BSA in PBS. Chamber slides were washed and incubated for 30 min with secondary anti-mouse antibody (biotinylated goat anti-mouse immunoglobulins; Dako, Glostrup, DK) diluted 1:200 in 1% BSA in PBS. Avidin-biotin detector complex (Vectastain ABC kit; Vector Laboratories, Burlingame, CA, USA) was applied for an incubation time of 30 min. Chambers have been removed from the glass base and were then incubated for approximately 4 min with 3, 3’diaminobenzide tetrahydrochloride (DAB chromogen/substrate kit; Diagnostic BioSystem, Pleasanton, CA, USA). Sections were counterstained with Papanicolau’s hematoxylin solution for 45 s. After dehydration in alcohol and clarification in Diaphane, slides were mounted. All steps were performed at room temperature and in the dark, except for the primary antibody incubation at 4 °C. Each experiment was performed in triplicate. For each marker negative control were included and run in parallel. Staining evaluation was performed with an optical microscope (Eclipse E600; Nikon, Shinjuku, Japan). The percentage of P-gp, BCRP, p53, TERT and Ki67 immunopositive cells, both for CIPp and CIPm, was calculated using the ‘Cell Counter Notice’ plug-in of ImageJ (US National Institutes of Health, Maryland, USA, 2018). For each marker and replicate of the experiment, a minimum of 1000 cells were evaluated when possible, images taken at optical microscope were analyzed using the NIS-Elements F image software (Nikon, Shinjuku, Japan). Cell percentages were averaged over triplicate samples, and the data are expressed as the mean ± SD.

### Multidrug Resistance Direct Dye Efflux Assay

The MDR Direct Dye Efflux Assay kit (Merck, Kenilworth, NJ, USA) was used to measure P-gp and BCRP activity following manufacturer’s instructions. CIPp and CIPm cells, seeded on T25 culture flasks, were detached by trypsinization, counted and 4 aliquots of 1 × 10^6^ cells for each cell lines were prepared. One aliquot for each cell line was employed as blank for flow cytometry analysis. Other cell suspensions were washed in DPBS and loaded with 0.2 μg/ml of 3,3′-Diethyloxacarbocyanine Iodide DiOC2(3) in Cold Efflux Buffer (RPMI + 2% BSA) overall named DiOC2(3) Loading Buffer. Specifically, samples were incubated with 1 ml of DiOC2(3) Loading Buffer /1 × 10^6^ cells for 15 min on ice to minimize efflux activity. After the incubation time, cells were washed twice in Cold Efflux Buffer (2.5 ml of Cold Efflux Buffer /1 × 10^6^ cells). Cell pellets were suspended in 3 ml of 3 different buffers: 37 °C Warmed Efflux Buffer (positive control - P); 37 °C Warmed Efflux Buffer with Vinblastine (V); 4 °C Ice Cold Buffer (negative control - N). DMSO was added as vehicle control. Samples in Warmed Efflux Buffer were incubated at 37 °C while samples in Ice Cold Buffer were maintained on ice for 15 min, 1 h, 4 h and 24 h. At the end of each incubation times, cells were washed twice in Cold Efflux Buffer then cell pellets were suspended in 0.5 ml of Cold PI (1:50) Buffer to evaluate cell mortality.

The samples were analysed with the MACSQuant Analyzer 10 (Miltenyi Biotec Bergisch Gladbach, Germany) equipped with three lasers (405, 488 and 638 nm). Data analysis was performed using Flowlogic software (Miltenyi Biotec, Bergisch Gladbach, Germany). Cellular events were discriminated from debris using forward (FSC-A) and side scatter (SSC-A). Doublets have been excluded from analysis by FSC-area and weight while dead cells were eliminated from the analysis basing on PI fluorescence. DiOC2(3) signal (excitation/emission: 482/500) was collected in the B1 channel (525/50 nm). For each analysis we recorded at least 200,000 events. Median fluorescence intensity (MFI) of each peak were considered so differences (Δ) between MFI values of sample maintained on ice and sample in warmed buffer (ΔN-P) and between sample in warmed buffer and sample added with Vinblastine (ΔP-V) were calculated. For spectral compensation, the following samples were utilized: cells not stained (Blank), cells loaded for 15 min on ice with DiOC2(3) Loading Buffer and cells stained with PI (1:50) in Cold Efflux Buffer. At the end of the incubation time cells were washed and suspended in 250 μl of Cold Efflux Buffer. The compensation setting was applied to all analysis.

### Statistical analysis

Statistics were calculated with Prism 7 software (GraphPad San Diego, CA, USA). Graphs were created by the same software.

#### EC50 determination

EC50(_20h_) values were calculated using nonlinear regression curve as previously described [79]. Each treatment was analysed in quadruplicate, and the experiment was repeated three times.

#### Cell cycle analysis

Data statistics were performed with Student’s t test comparing the percentage of cells in every phase of cell cycle (subG0/G1, G0/G1, S, G2/M) in treated vs control cells at the same time points (3, 6, 12 and 20 h). *p* < 0.05 was considered significant.

#### Gene expression

Data statistics were performed with Student’s t test comparing treated vs control at the same time points (3, 6, 12, 20 and 48 h). *p* < 0.05 was considered significant.

#### Cell immunocytochemistry

Data statistics were performed with the two-tail Fisher exact test comparing the percentage of positive cells between controls and DOX treated at each time point (12, 24, 48 h). *p* < 0.05 was considered significant.

#### Multidrug Resistance Direct Dye Efflux Assay

Data statistics were performed by Student’s t test on median fluorescence intensity (MFI) values comparing the two cell lines in the same condition (amount of fluorescent dye effluxed and reduction of dye effluxed in presence of Vinblastine at 15 min and 1, 4, 24 h). *p* < 0.05 was considered significant.

## Supplementary Information

**Additional file 1.** Cell cycle distribution in CIPp. Grouped histograms graph for CIPp treated with DOX EC50_(20h)_ for 3, 6, 12 and 20 h in comparison with the relative CTRs. Cell percentages were averaged over triplicate samples, and the data are expressed as the mean ± SD. Paired Student’s t-test (parametric data), asterisk indicates a significant difference (*p* < 0.05) between controls and treated cells. Sub G0/G1 blue, G0/G1 red, S yellow, G2/M green.

**Additional file 2.** Cell cycle distribution in CIPm. Grouped histograms graph for CIPm treated with DOX EC50_(20h)_ for 3, 6, 12 and 20 h in comparison with the relative CTRs. Cell percentages were averaged over triplicate samples, and the data are expressed as the mean ± SD. Paired Student’s t-test (parametric data), asterisk indicates a significant difference (*p* < 0.05) between controls and treated cells. Sub G0/G1 blue, G0/G1 red, S yellow, G2/M green.

**Additional file 3.** Western Blot for BCRP and P-gp. Canine liver was used as the positive control and canine skeletal muscle as negative control. The α-tubulin was used as a housekeeping protein. (A) Western Blot for BCRP detected a single molecular weight specific band of approximately 75 kDa. (B) Western Blot for P-gp detected a single molecular weight specific band of approximately 140 kDa. Lane 1 = 10 μg of liver tissue Lane 2 = 30 μg of liver tissue Lane 3 = 10 μg of skeletal muscle tissue Lane 4 = 30 μg of skeletal muscle tissue MWM = Molecular Weight Marker expressed in kDa.

**Additional file 4.** Immunocytochemistry (ICC) results and statistical comparisons. P-gp, BCRP, p53, TERT expression and the Ki67 proliferation index were evaluated in CIPp and CIPm by ICC at 12, 24, 48 h of treatment with the EC50_(20h)_ dose of DOX and compared to the corresponding control cultures.

## Data Availability

Elaborated data generated or analyzed during this study are included in this published article and its supplementary information files.

## Abbreviations

BCRP: Breast Cancer Resistance Protein
CMC: Canine mammary tumour
CTR: Control
DOX: Doxorubicin chlorohydrate
FBS: Foetal bovine serum
EC50_(20h)_: Median effective concentration at 20 h of DOX treatment
EDTA: Ethylenediaminetetraacetic acid
ICC: Immunocytochemistry
mRNA: Messenger Ribonucleic Acid
MTT: 3-(4,5-dimethylthiazol-2-yl)-2,5-diphenyltetrazolium bromide
p53: Tumour protein p53
PI: Propidium iodide
qRT-PCR: Quantitative Real Time Polymerase Chain Reaction
PBS: Phosphate-buffered saline
P-gp: P-glycoprotein
SDS: Sodium dodecyl sulphate
TERT: Telomerase reverse transcriptase
