# Supplementary figures and images for: Doxorubicin treatment modulates chemoresistance and affects the cell cycle in two canine mammary tumour cell lines
Source: BMC Vet Res. 2021 Jan 18;17:30. doi: 10.1186/s12917-020-02709-5 (PMC7814552; doi:10.1186/s12917-020-02709-5)

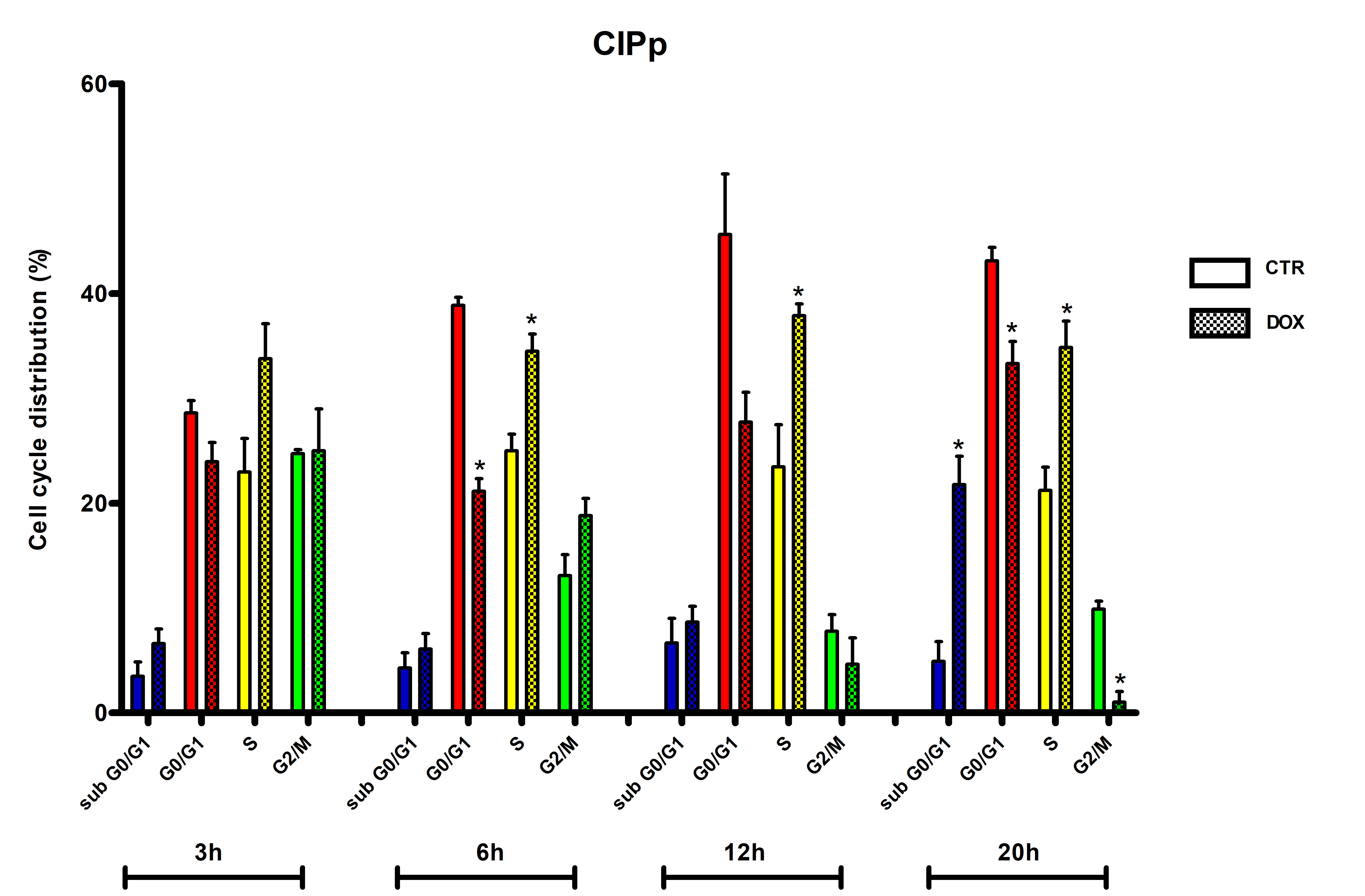

Supplement: Supplementary file 1 — Additional file 1. Cell cycle distribution in CIPp. Grouped histograms graph for CIPp treated with DOX EC50(20h) for 3, 6, 12 and 20 h in comparison with the relative CTRs. Cell percentages were averaged over triplicate samples, and the data are expressed as the mean ± SD. Paired Student’s t-test (parametric data), asterisk indicates a significant difference (p < 0.05) between controls and treated cells. Sub G0/G1 blue, G0/G1 red, S yellow, G2/M green. [file 12917_2020_2709_MOESM1_ESM.jpg]

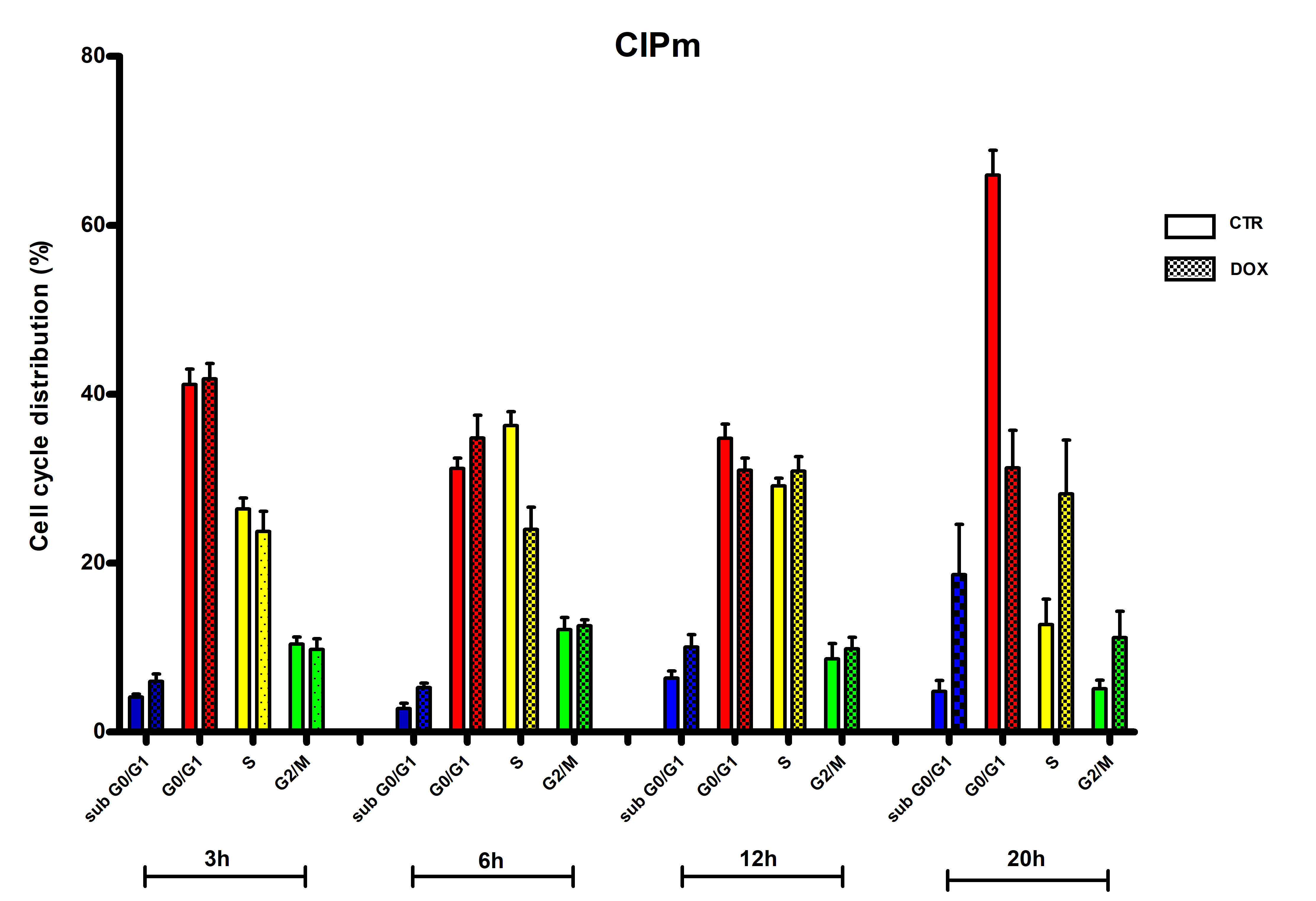

Supplement: Supplementary file 2 — Additional file 2. Cell cycle distribution in CIPm. Grouped histograms graph for CIPm treated with DOX EC50(20h) for 3, 6, 12 and 20 h in comparison with the relative CTRs. Cell percentages were averaged over triplicate samples, and the data are expressed as the mean ± SD. Paired Student’s t-test (parametric data), asterisk indicates a significant difference (p < 0.05) between controls and treated cells. Sub G0/G1 blue, G0/G1 red, S yellow, G2/M green. [file 12917_2020_2709_MOESM2_ESM.jpg]

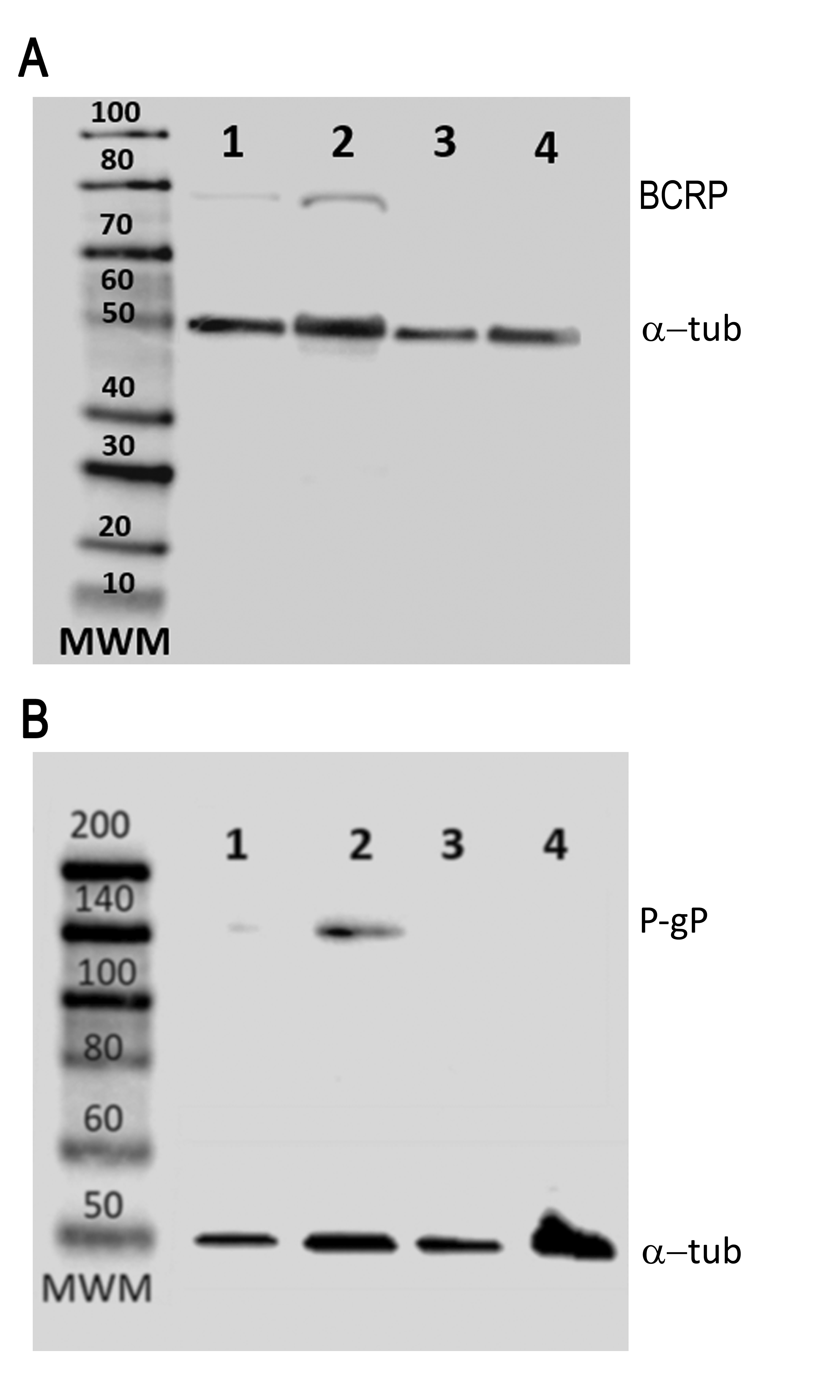

Supplement: Supplementary file 3 — Additional file 3. Western Blot for BCRP and P-gp. Canine liver was used as the positive control and canine skeletal muscle as negative control. The α-tubulin was used as a housekeeping protein. (A) Western Blot for BCRP detected a single molecular weight specific band of approximately 75 kDa. (B) Western Blot for P-gp detected a single molecular weight specific band of approximately 140 kDa. Lane 1 = 10 μg of liver tissue Lane 2 = 30 μg of liver tissue Lane 3 = 10 μg of skeletal muscle tissue Lane 4 = 30 μg of skeletal muscle tissue MWM = Molecular Weight Marker expressed in kDa. [file 12917_2020_2709_MOESM3_ESM.jpg]
